# Complicated Sporadic Cardiac Myxomas: A Second Recurrence and Myxomatous Cerebral Aneurysms in One Patient

**DOI:** 10.1155/2013/642394

**Published:** 2013-12-25

**Authors:** Mazen E. Iskandar, Kamellia Dimitrova, Charles M. Geller, Darryl M. Hoffman, Robert F. Tranbaugh

**Affiliations:** ^1^Department of Surgery, Beth Israel Medical Center, 350 East 17th Street 16 Baird Hall, New York, NY 10003, USA; ^2^Department of Cardiac Surgery, Beth Israel Medical Center, 317 East 17th Street, New York, NY 10003, USA

## Abstract

A second recurrence of an excised nonfamilial cardiac myxoma is rare. Myxomatous cerebral aneurysms as a complication of cardiac myxomas are equally rare. A unique case of a patient with a total of 4 myxomas over a 20-year interval is presented. Her most recent presentation was a second recurrence of a left atrial myxoma, a de novo right atrial myxoma, and multiple cerebral myxomatous aneurysms. The challenging reconstruction of the normal anatomy was achieved with the use of porcine extracellular matrix patches. A diagnostic cerebral angiogram was later performed, and the aneurysms will be monitored for growth and possible intervention.

## 1. Introduction

Although it is a histologically benign disease, it is not uncommon for a cardiac myxoma to recur after surgical therapy. Recurrent myxomas could be classified into four etiologic groups: inadequate resection, totipotent multicentricity, familial type, and metastatic recurrence [1]. Most recurrences are attributed to multicentric disease rather than inadequate resection or seeding of tumor cells during the primary resection. The frequency of recurrence is higher in patients with a familial predisposition such as the Carney complex. Second recurrences have rarely been reported and most have been in the familial setting [2, 3].

Myxomatous emboli are rare events that can affect any arterial system including the intracerebral circulation. Aneurysmal growth of the intracerebral arteries secondary to myxomatous emboli is well described and difficult to treat [4]. The presence of a second recurrence of a cardiac myxoma and a simultaneous myxomatous intracerebral aneurysm in the same patient has not been previously reported. A staged approach to such a patient's management with the use of a porcine extracellular membrane to reconstruct the left and right atria along with the interatrial septum is described.

## 2. Case Presentation

**Figure 1:**
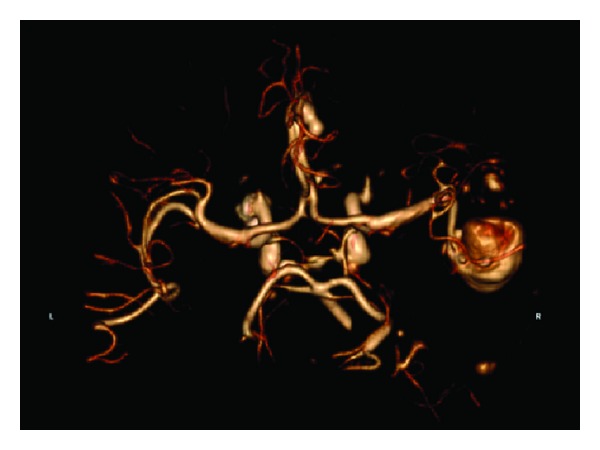
CT angio of the head showing two myxomatous fusiform aneurysms in the right middle (2.9 cm × 1.8 cm) and anterior cerebral arteries (10 mm × 5 mm).

**Figure 2:**
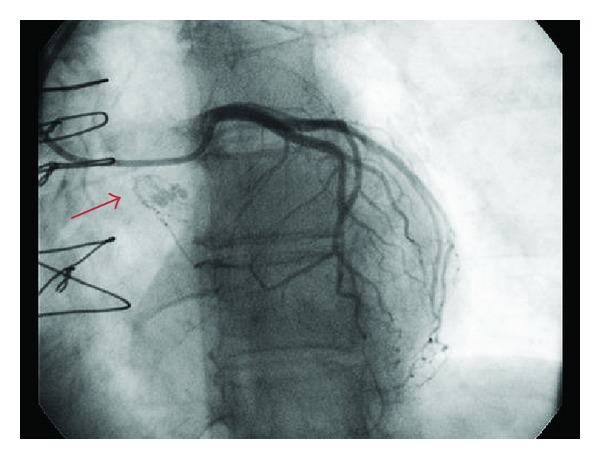
Cardiac catheterization showing normal coronaries and a tumor blush.

A 69-year-old diabetic and hypertensive woman presented in July 2012 with a complaint of left arm numbness, weakness, and dysarthria. She had 2 previous left atrial myxomas resected and had presented both times with similar transient ischemic attacks. In June 1992, she underwent wide excision of a pedunculated 2.3 cm friable left atrial myxoma arising from the lateral left atrial wall above the right inferior pulmonary vein orifice, and autologous pericardium was used for reconstruction. In June 1997, she had a complete excision of a broad-based 2.0 cm friable left atrial myxoma arising between the left atrial appendage and the A1 section of the mitral valve. The resulting endothelial defect was closed primarily, and the margins were again negative. Her family history was negative for cardiac tumors. On physical exam she had a left facial droop, 4/5 motor power in her left arm, and no cardiac murmurs or visible skin lesions. A head MRI showed an acute right parietal stroke, and a CT angiogram (Figure 1) was consistent with two myxomatous fusiform aneurysms in the right middle (2.9 cm × 1.8 cm) and anterior cerebral arteries (10 mm × 5 mm). A transesophageal echocardiogram was obtained revealing a recurrent left atrial myxoma arising from the interatrial septum. Cardiac catheterization (Figure 2) showed normal coronary arteries and a tumor blush in the area of the left atrium. Preoperative chest CT scan demonstrated that the heart and aorta were not adjacent to the sternum thus allowing safe sternal reentry for the third time.

A redo sternotomy was planned to resect the recurrent myxoma followed by cerebral angiography. Standard cardiopulmonary bypass and cardioplegic arrest were employed. A right atriotomy revealed an incidental smooth 1.5 cm flat right atrial mass that was excised. Frozen section confirmed the suspicion of a myxoma. A left atriotomy incision was next made, and the interatrial septum containing a gelatinous 2.0 × 1.5 cm friable mass, the lateral free left atrial wall extending to the right pulmonary veins, and the free right atrial wall were excised. Reconstruction was done using 2 porcine extracellular matrix (CorMatrix ECM, Roswell, Georgia) patches. The first patch replaced the interatrial septum and the anterior wall of the left atrium laterally to the orifices of the right pulmonary veins. The second triangular-shaped ECM patch closed the right atriotomy and extended from the medial aspect of the right atriotomy to the pulmonary vein-ECM suture line. The patient did well and was discharged on the 5th postoperative day. A review of all previous and current specimens showed that the 4 myxomas were completely excised and identical, corresponding to a polypoid benign myxoma extending from the endocardial surface.

The cerebral myxomatous aneurysms were approached by a multidisciplinary team consisting of neurologists, interventional radiologists, and neurosurgeons. A diagnostic cerebral angiogram was performed 5 months later, and the aneurysms were found to be fusiform, such that coil embolization could result in total occlusion of the affected vessels. The recommendation was to monitor with serial CT scans. The alternative, a bypass with coiling, was thought to be of high risk and would only be indicated if the aneurysms displayed growth on surveillance. Repeat head CT 3 months later showed the presence of a thrombus in the right MCA aneurysm without any significant increase in size, and the patient will be imaged again in 3 months.

## 3. Discussion

Recurrent myxomas are not uncommon with recurrence rates approaching 7% in nonfamilial cases and as high as 21% in familial cases [2, 3]. Second recurrences, however, are exceptionally rare and have been reported in a small number of both familial and nonfamilial patterns [2, 3]. It is unlikely that incomplete resection or malignant transformation accounts for few if any of recurrent tumors. Our patient had 3 left atrial myxomas, all in different intra-atrial locations confirming that incomplete resection was not the cause of her recurrent myxomas. Familiar occurrence or totipotent multicentric tumors account for most of recurrent observed myxomas in the current era. Our patient appears to have a totipotent multicentric myxoma with 3 different left atrial locations and a separate right atrial origin.

Myxomatous cerebral aneurysms are a rare manifestation of tumor embolization to the cerebral arterial system, and only 25 cases have been reported since 1990 [4]. It is thought that tumor emboli deposit in the arterial wall causing endothelial damage, connective tissue proliferation, and an inflammatory reaction leading to a fusiform dilatation of the vessel [4]. Cerebral aneurysms are treated with clipping or endovascular coiling if they are saccular, and embolizing them does not obstruct blood flow to prevent rupture and subarachnoid hemorrhage.

Our approach was to address the recurrent myxoma first due to the recent, acute embolic stroke. A third sternotomy was uneventful, and complete wide excision of both the right and left atrial myxomas was done. The atria were reconstructed using CorMatrix extracellular membrane. The use of ECM has been well described in intracardiac repair [5]. Once the ECM is repopulated with myocardial cells, there is the theoretical advantage of having functioning atrial tissue given the large patches required.

## 4. Conclusion

A patient with a total of 4 geographically independent myxomas over 20 years and cerebral myxomatous aneurysms was presented. The etiology of the recurrence is likely due to a totipotent multicentric etiology. Porcine extracellular matrix extends the armamentarium of options available for reconstruction in complex redo cases.
